# Need for standardization in sub-lethal antibiotics research

**DOI:** 10.3389/fmicb.2023.1299321

**Published:** 2023-12-21

**Authors:** Fabian Thurner, Fatima AlZahra’a Alatraktchi

**Affiliations:** Department of Science and Environment, Roskilde University, Roskilde, Denmark

**Keywords:** sub-lethal antibiotics, MIC, sub-MIC, MSC, MIPC, virulence, standardization

## Abstract

While monitoring and managing resistant and persistent microbes is of utmost importance and should not be glossed over, one must also focus on mitigating the microbe’s ability to cause harm. Exploring the concept of lowering or even suppressing the microbe’s virulence with sub-Minimum Inhibitory Concentration (MIC) antibiotics holds promise and warrants further investigation. At present, such antibiotic concentrations have mostly been studied to cover the side-effects of gradient exposure, overlooking the possibility of utilizing them to influence not only bacterial virulence, but also colonization, fitness and collateral sensitivities. This review focuses on conflicting findings of studies demonstrating both increased and decreased virulence in microbes under sub-MIC antibiotic exposure. It identifies lack of standardization in this field of research as one of the main culprits for discordant results across numerous studies on virulence. It critically discusses important terminology related to bacterial traits and existing methods to determine MIC and sub-MIC ranges. Lastly, possible directions toward standardized sub-MIC profiling and thereby tailored treatment options in the future are explored.

## 1 Introduction

Antibiotics represent a curse and blessing at the same time. While their positive effects on global welfare are undeniable, the emergence of antimicrobial resistance (AMR) undoubtably poses severe challenges for the health care sector. Especially multidrug resistant (MDR) pathogens such as methicillin-resistant *Staphylococcus aureus* (MRSA) cause havoc in hospital environments around the globe (Turner et al., 2019). Moreover, persistent bacteria contribute to long term, recurrent infections that are tough to treat (Madhusoodanan, 2022). Combined efforts are being made to mitigate AMR and therefore lower the clinical burden on a global scale.

At present, to elucidate the dynamics of resistance evolution, sub-lethal antibiotic concentrations have largely been studied in the context of gradient exposure (Lagator et al., 2021). Thus, the possibility of harnessing sub-lethal doses of antibiotics to impact virulence, colonization, fitness and collateral sensitivities has not received sufficient attention. In particular the possibility to lower or even suppress bacterial virulence is not yet a well-recognized path, although ultimately, it is not the resistance but the virulence which causes disease (Laxminarayan et al., 2013). Evidence is increasing that pathogenic transcripts can be affected by low antibiotic concentrations below the Minimum Inhibitory Concentration (MIC) (Chen et al., 2021; Nolan and Behrends, 2021). Our own research demonstrated that toxin production in environmental *Pseudomonas aeruginosa* isolates changes when exposed to sub-inhibitory antibiotic concentrations (Mojsoska et al., 2021).

**Figure 1:**
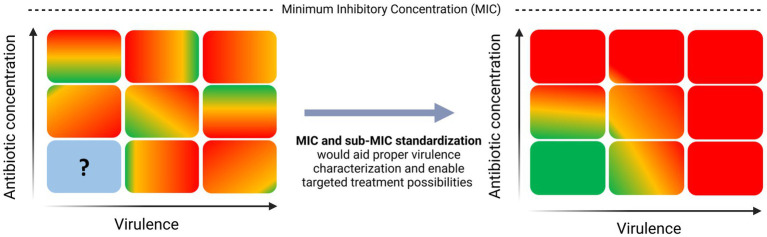
Need for standardization in sub-MIC research to aid virulence characterization in pathogenic bacteria.

Current research on the effect of sub-MIC antibiotics on virulence points in many directions, making it tough to draw comparative and informed conclusions. In this review, we propose the term Virulence Inhibiting Concentration (VIC) as the potential sub-MIC antibiotic concentration inhibiting virulence of a given pathogen. We believe that standardization of MIC and sub-MIC zone methodologies would contribute to the determination of the VIC of various pathogens (Figure 1). To prove this claim, this paper highlights how different methods for MIC profiling can lead to contradictory results across virulence studies using the same reference strains. However, we also acknowledge that other contributing factors such as strain-specific features like mutations in regulatory systems or specific environmental adaptation mechanisms that impact virulence regulation cannot be ruled out in other studies that use different strains. Thus, lack of standardization in MIC and sub-MIC determination is solely one piece of the puzzle and future efforts need to be directed toward fully understanding the impact of sub-lethal antibiotics on virulence. Lastly, we aim to give an overview of what needs to be taken into consideration when taking on the challenge to standardize sub-MIC research.

## 2 Characterizations of bacterial resistance, tolerance, and persistence

AMR is a global public health “ticking bomb.” Predictions estimate that by 2050, the annual global gross domestic product (GDP) will see a decline of 1.1–3.8% relative to a base-case scenario assuming no AMR effects. This shortfall is projected to reach costs of USD 1 trillion to USD 3.8 trillion per year after 2030 (OECD, 2018). Among natural reasons such as genetic mutation and acquisition of resistance conferring genes via horizontal gene transfer (HGT), rise in AMR can primarily be attributed to the overuse of antibiotics in medicine and agriculture and the spread of resistant microorganisms in the environment, particularly in low-income countries with poor sanitation, allowing for food and drinking water contamination (Blair et al., 2015; Von Wintersdorff et al., 2016; Bastaraud et al., 2020; Uddin et al., 2021; Urban-Chmiel et al., 2022). Diseases caused by resistant pathogens are notoriously hard to treat and the amount of novel antibiotics able to eradicate them is near zero: The World Health Organization (WHO) reports that in the 2021 clinical pipeline of 45 novel antibiotics only two are active against at least one MDR bacterium from the “critical” category (World Health Organization, 2021). Next to AMR, the emergence of drug-tolerant subpopulations of microbes exacerbates the situation by causing recurrent infections in the host (Madhusoodanan, 2022).

**Figure 2:**
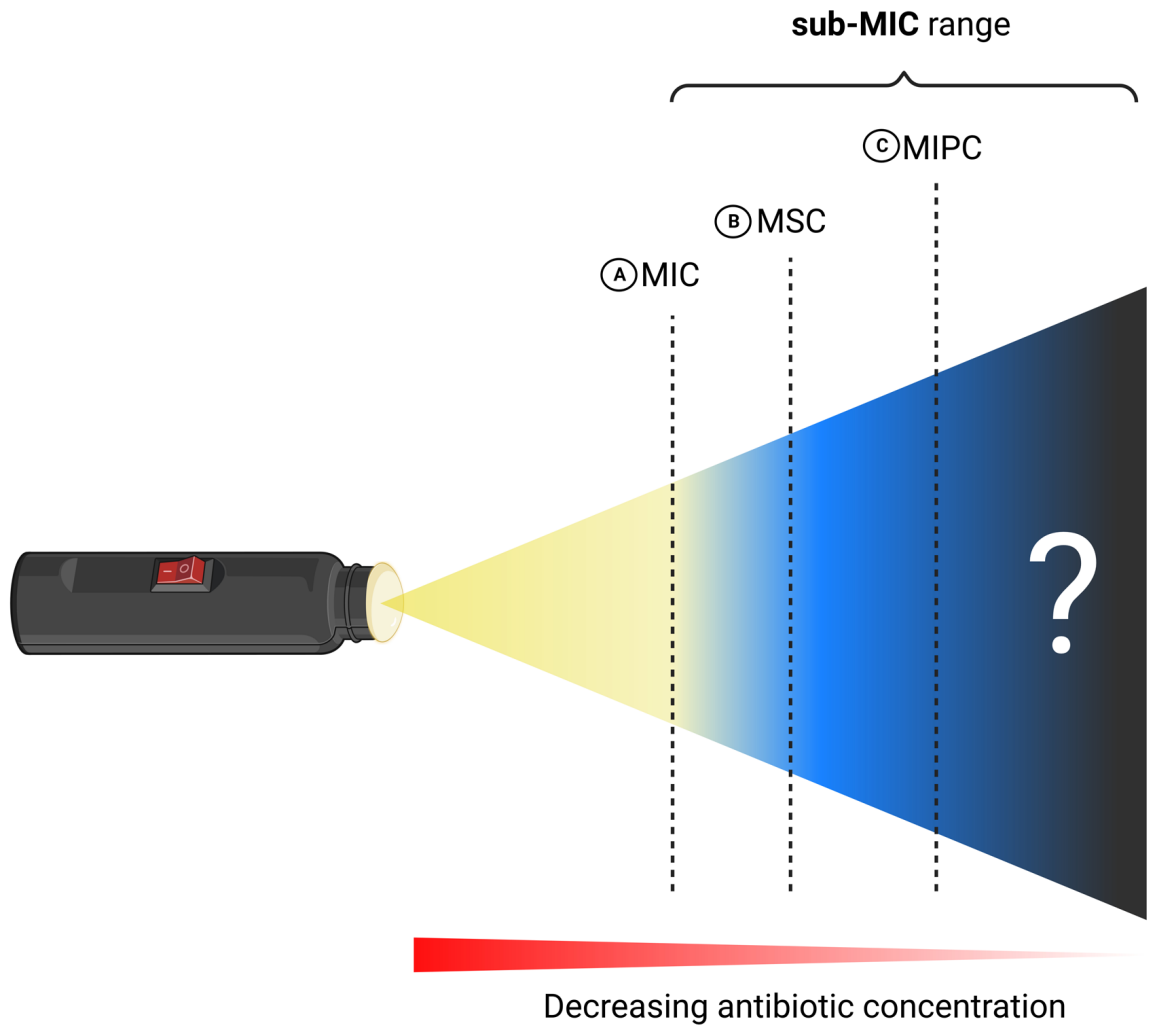
Schematic illustration of MIC and sub-MIC zones. **(A)** Minimum Inhibitory Concentration (MIC). **(B)** Minimum Selective Concentration (MSC) and **(C)** Minimum Increased Persistence Concentration (MIPC). Created with BioRender.com.

It was as early as 2000 that the concept of a “selective window” at which selection of resistance conferring genes is strongest had been formally proposed (Negri et al., 2000). There, the first experimental evidence of selection of low-level antibiotic resistant genetic variants is presented. Later in 2011, pioneering studies introduced the terminology Minimal Selective Concentration (MSC) and specified a sub-MIC window where the fitness cost of the resistance is balanced by the antibiotic-conferred selection for the resistant mutant (Figure 2) (Gullberg et al., 2011; Liu et al., 2011). Subsequently, key-literature exploring selection dynamics at sub-MIC antibiotic levels has been published (Hughes and Andersson, 2012; Andersson and Hughes, 2014; Gullberg et al., 2014; Lundström et al., 2016; Khan et al., 2017; Kraupner et al., 2018; Wistrand-Yuen et al., 2018). It was not before 2020 that a sub-MSC window selective for persisters, termed the minimal increased persistence concentration has been proposed (MIPC, Figure 2) (Stanton et al., 2020). Since MSC and MIPC have been delineated based on microbial traits such as resistance and persistence, we believe it imperative to have a fundamental understanding of these terms in order to fully comprehend the sub-MIC zones.

MIC represents the lowest concentration of a given antibiotic that inhibits microbial growth. Since an increase in resistance is reflected in an increase in MIC, it can be used to give insight into a pathogen’s resistance. MIC values are dependent on many factors such as microbial strain, type of antibiotic and method of MIC determination and are widely used in clinical settings to quickly assess resistance in bacterial isolates. Here, it is important to mention the concept of heteroresistance, a phenomenon describing a bacterial isolate that contains sub-populations of cells displaying a significantly higher MIC (Andersson et al., 2019; Balaban et al., 2019). An excellent paper by Andersson et al. further goes into detail and outlines the factors to consider when studying heteroresistance, while focusing on mechanistic details and clinical relevance of the transient phenomenon (Andersson et al., 2019).

Tolerant cells can survive but not grow under antibiotic exposure many times the MIC (Kester and Fortune, 2014). Notably, due to the inherent nature of bacteriostatic antibiotics to inhibit growth, the definition of tolerance can only be applied in the context of bactericidal antibiotics. The special phenomenon when subpopulations with different tolerance levels coexist within the same clonal population is called persistence, a term coined by Joseph Bigger in 1944 (Bigger, 1944; Kester and Fortune, 2014; Brauner et al., 2017; Balaban et al., 2019). Mainly attributed to epigenetics, persistent pathogens manage to increase their survival in the presence of antibiotics without resistance-conferring genes (Riber and Hansen, 2021). This phenomenon arises from reprogramming the transcriptional landscape and allows for establishment of dormant cell states. When in dormancy, such cells downregulate their metabolism and do not divide and are therefore not vulnerable to antibiotics affecting cell division (Riber and Hansen, 2021).

The standard staring point to evaluate tolerance/persistence is through performance of time-kill assays and generation of characteristic biphasic killing curves. However, these do not always reflect persistence, since a similar curve can arise from resistant mutants in the sample population. To rule this out, the surviving bacteria should be regrown under the same antibiotic conditions. If resistance was the major driver of the biphasic curve observed, a much higher proportion of the population will show reduced killing in the second assay. If persistence caused the biphasic killing curve in the first place, no change in killing should be observed (Balaban et al., 2019). Moreover, since persistence can easily be mistaken for transient phenomena such as heteroresistance, it is advised to perform the assays at substantially high concentrations of antibiotics. In persisters, only a weak dependence of antibiotic concentration on killing is expected, whereas strong correlation hints at resistance mechanisms in play (Balaban et al., 2019). However, as time-kill curves rarely follow strict exponentiality it is impossible to quantify tolerance and persistence and the killing rate cannot be compared across different strains and growth conditions. Moreover, time-kill assays are labor intensive and thus rarely utilized in the health care sector (Brauner et al., 2017). Therefore, Brauner et al. developed a novel method to quantify tolerance without the need to perform time-kill assays. They propose clinical implementation of a simple timescale parameter: The “minimum duration for killing 99% of the population” (MDK_99_) (Brauner et al., 2017). The MDK can be easily determined by exposing populations of bacteria to different antibiotic concentrations for varied time periods, subsequently evaluating the presence or lack of survivors (Brauner et al., 2017).

## 3 Impact of sub-lethal antibiotic concentrations on virulence are yet to be explored

Virulence represents the ability of a pathogen to cause harm in the infected host. Virulence factors can lead to improved adhesion, evasion of the hosts’ immune system or production of toxins, all of which aid harmful microbes to colonize the host at a cellular level (Sharma et al., 2017). One notorious example of a pathogen that uses toxins as virulence factors is *Bacillus anthracis* which establishes systemic infection by using two lethal toxins termed “lethal factor” and “edema factor” (Sharma et al., 2017). Pyocyanin is another example of a toxin that has been proven to have great implications in the chronic nature of pseudomonal infections (Hall et al., 2016; Alatraktchi et al., 2020).

Another determinant of virulence is biofilm formation in pathogens such as *Staphylococcus aureus* and *Pseudomonas aeruginosa.* It acts as a physical barrier against the host defense systems (Sharma et al., 2023). Furthermore, the virulence factor alginate has been shown to play an essential role in thick biofilm formation as well as protection of the pathogen against the hosts by shielding against antibiotics, neutralizing reactive oxygen species and counteracting macrophagic uptake (Simpson et al., 1988; Yu et al., 1995; Skariyachan et al., 2018). Lastly, biofilm formation has major clinical implications in chronic infections and has been shown to be mechanically linked to emergence of persisters (Pan et al., 2023).

Sub-lethal antibiotic exposure evidently alters bacterial virulence and the underlying mechanisms by which the antibiotic concentrations modify various virulence traits are multifaceted and complex. In a paper from 2006, Linares et al. illuminates this area of research by pointing out that sub-MIC antibiotics can also act as signaling agents instead of weapons (Linares et al., 2006). By using a combination of genomic and functional assays they demonstrate that sub-lethal levels of Tobramycin, Tetracycline and Norfloxacin influences virulence in *Pseudomonas aeruginosa.* Low doses of these antibiotics increased the bacterium’s ability to colonize potential hosts by enhancing biofilm formation and motility. Moreover, sub-MIC Tetracycline was shown to trigger the type III section system, leading to enhanced cytotoxicity of the bacterium (Linares et al., 2006). Another example of how sub-MIC antibiotics can act as signals can be found in a study by Shang et al., who revealed that some β-lactam antibiotics promote expression of a cluster of lipoprotein-like genes that consequently enhance virulence of MRSA (Shang et al., 2019; Chen et al., 2021). Lastly, the antibiotic Fosfomycin was shown to bind to Lys154 and Asp108 of the α-toxin of *Staphylococcus aureus,* thereby inhibiting its activity (An et al., 2019).

Researchers have investigated the effect of different sub-MIC concentrations of various antibiotics on selected virulence traits of a variety of bacterial species and associated strains. In an excellent review, the results of such studies focusing on virulence in *Pseudomonas aeruginosa* have been consolidated (Nolan and Behrends, 2021). They identified a common trend that most sub-MIC antibiotics reduce the *in vitro* virulence of *Pseudomonas aeruginosa* (Nolan and Behrends, 2021). Furthermore, they conclude that that so far, the virulence of planktonic *Pseudomonas aeruginosa* has been assessed mostly over a short time (<24 h antibiotic exposure). They stress the point that results obtained in such regulated, short-term single strain assays are not necessarily translatable to the pathogens’ *in vivo* virulence (Nolan and Behrends, 2021). Another exhaustive review summarizes the impact of sub-MIC antibiotic exposure on virulence in *Staphylococcus aureus* (Chen et al., 2021). They state that manifestations of effects on virulence of sub-MIC antibiotics points into different directions, dependent on the virulence profile, growth stage and culture conditions of *Staphylococcus aureus* at the time of antibiotic administration (Chen et al., 2021).

A study from 2021 by Davarzani et al. investigated the effect of sub-MIC concentrations of the antibiotic Gentamicin on alginate and biofilm production in *Pseudomonas aeruginosa*. They found that alginate and biofilm production under exposure of 1/2 MIC and 1/4 MIC was either significantly up- or downregulated to various degrees depending on the clinical isolate. The MIC values of Gentamicin for the clinical isolates P1, P2, P3 and for the two reference strains 8821 M and PAO1 have been calculated to be 0.25, 0.25, 1, 2 and 0.5 μg/mL, respectively (Davarzani et al., 2021).

An excellent study investigated the effects of sub-inhibitory concentrations of quinolones, aminoglycosides, β-lactams and macrolides on alginate production in *Pseudomonas aeruginosa* (Majtán and Hybenová, 1996). Some antibiotics such as Enoxacin or Nalidixic acid followed a discernible pattern of decreasing alginate production with increasing sub-MIC concentration. Others, such as Ciprofloxacin showed significant upregulation of alginate at low concentrations 1/16 MIC (50 μg/mL) but downregulation at 1/4 MIC (190 μg/mL) (Majtán and Hybenová, 1996).

A sub-MIC study from Molinari et al. concludes that production of pseudomonal virulence factors like pyocyanin, various exotoxins such as elastases and proteases and pathogenic behavior like motility is highly strain-, antibiotic- and concentration-dependent (Molinari et al., 1993). Notably, they were able to achieve unambiguous results for Azithromycin which inhibited pyocyanin production in all 10 strains tested.

Furthermore, Mojsoska et al. investigated the effect of sub-MIC concentrations of Ciprofloxacin, Tobramycin and Meropenem on pyocyanin production in environmental *Pseudomonas* strains (Mojsoska et al., 2021). Their results point out that virulence in *Pseudomonas* is both dependent on the strain and the antibiotic used: Tobramycin significantly downregulated levels of pyocyanin in the Pae112 strain whereas in PAO1 levels remained unchanged. On the other hand, Ciprofloxacin caused upregulation of virulence across all strains tested (Mojsoska et al., 2021).

While it is clear that sub-lethal antibiotics have great impact on bacterial virulence, the extent and general direction of it remains mostly unclear. We acknowledge that many factors such as the genetic background of the strains can contribute to the ambiguous results. Nonetheless, we claim that with standardization in MIC determination and consequently more accurate determination of sub-lethal antibiotic concentrations, bacterial virulence can generally be better understood, and the proposed VIC could be better determined. The following section aims to give examples of how initial MIC determination across studies using the same reference strains differs and how this makes it impossible to properly compare sub-MIC concentrations and their impact on bacterial traits such as virulence.

### 3.1 Variability in MIC determination contributes to inconsistent sub-MIC calculations across studies

**Figure 3:**
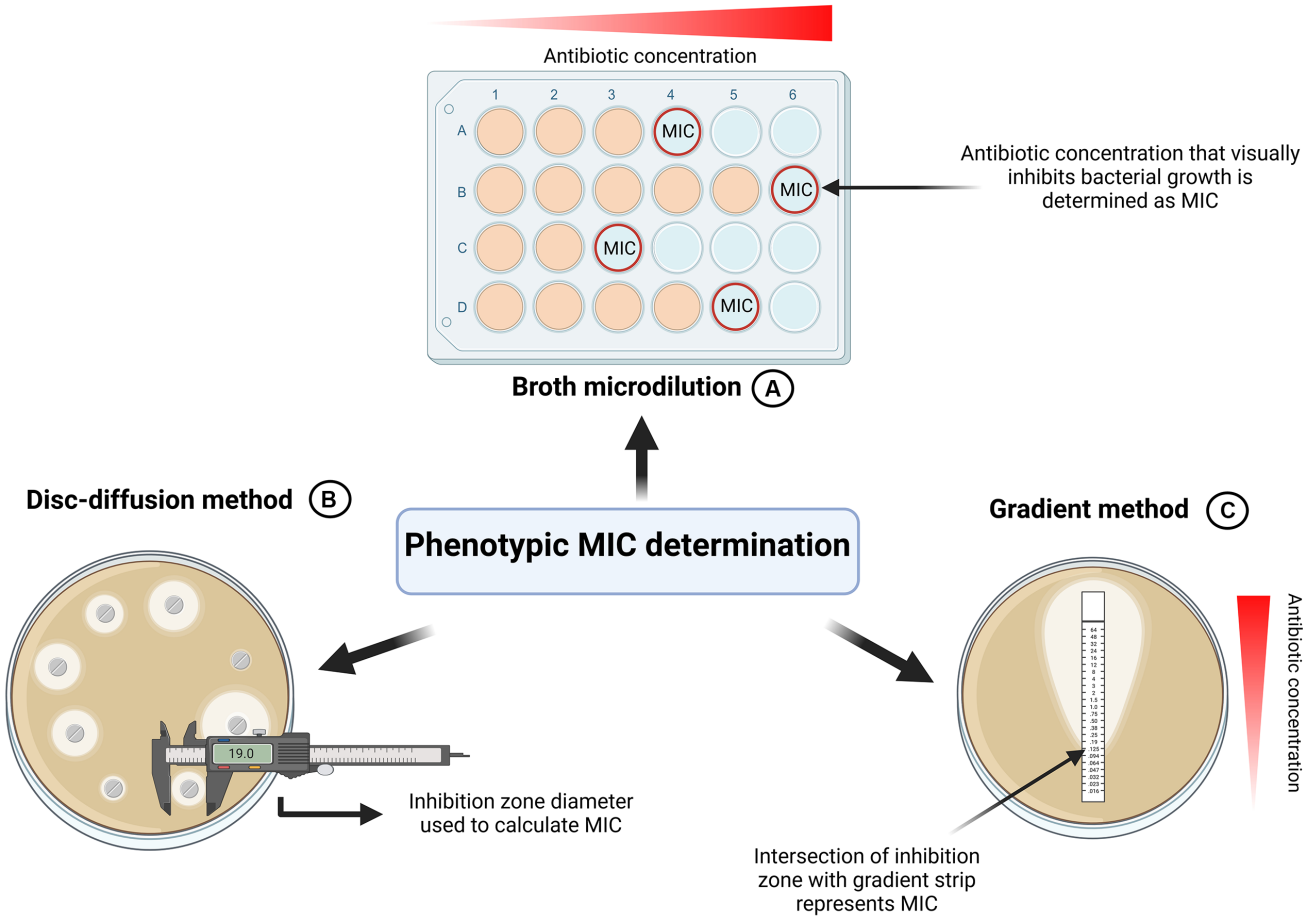
Graphical summary of phenotypic MIC determination. **(A)** Broth Microdilution. Standardized by EUCAST and CLSI (EUCAST, 2022; CLSI, 2023). Most commonly used method for MIC determination. **(B)** Disk-diffusion method (EUCAST, 2023). MIC is determined based on inhibition zone parameter. **(C)** Gradient method. Concentration at which the inhibition zone intercepts the strip represents MIC. Created with BioRender.com.

To detect antibiotic resistances in bacterial isolates as well as to find the right drug to treat infections, antibiotic susceptibility testing has become an integral part of the health care and research sector (Jorgensen and Ferraro, 2009). The metric to quantify resistance is termed Minimum Inhibitory Concentration (MIC). The European Committee for Antimicrobial Susceptibility Testing (EUCAST) defines it as the lowest concentration “that, under defined *in vitro* conditions, prevents the growth of bacteria within a defined period of time”(EUCAST, 2000). Next to the EUCAST framework, CLSI represents another established organization describing MIC determination in standardized ways (CLSI, 2023). Various phenotypical-, molecular- and mass spectrometry-based methods for MIC determination have been described (Gajic et al., 2022). As investigating the entirety of methods would be outside the scope of this review paper, it will instead focus on three more frequently used and accessible methods in routine clinical microbiology: “Microbroth dilution,” “disk-diffusion” and “gradient test” (Figure 3).

To precisely calculate various sub-MIC concentrations, it is imperative to properly determine the MIC of the antibiotics of interest in the first place. Since standardized frameworks are available this might seem like a simple task to achieve. However, this review identified various discrepancies in MIC determination across numerous studies, ultimately contributing to different MIC values and consequently, incomparable results. For antimicrobial susceptibility testing of rapidly growing aerobic bacteria, EUCAST and CLSI recommend the broth microdilution (EUCAST, 2022; CLSI, 2023). To cultivate microorganisms, un-supplemented cation-adjusted Mueller-Hinton (MH) broth or agar is used for non-fastidious organisms. For fastidious organisms EUCAST stipulates the use of cation-adjusted MH broth or agar supplemented with 5% lysed horse blood and 20 mg/L B-NAD (EUCAST, 2022). In the broth microdilution method, a defined number of bacteria is inoculated in increasing concentrations of an antibiotic of choice. According to established organizations, the growth should be assessed visually by the unaided eye: Microbial growth can manifest both as turbidity of the media or as a visible deposit of cells at the bottom of the well. The absence of both cloudiness and cell deposits indicates lack of growth and determines the MIC value (EUCAST, 2022) (Figure 3A).

Although most published studies investigating the impact of sub-MIC antibiotics perform the broth microdilution for the initial MIC determination, changes in parameters such as media used for the dilution drastically alter the outcome of MIC levels. A striking example of this are three papers investigating sub-MIC effects of Gentamicin on virulence in *Pseudomonas aeruginosa,* each using different media. Davarzani et al. determined the MIC of Gentamicin for the reference strain PAO1 as 0.5 μg/mL, Khan et al. reported it to be 8 μg/mL and Marr et al. calculated a value between 1 and 2 μg/mL (Marr et al., 2007; Khan et al., 2020; Davarzani et al., 2021). Those three independent studies used MHB, Tryptic Soy Broth (TSB) and Lysogeny Broth (LB), respectively. Sometimes, the change in calculated MIC can be minor: Comparing the calculated MIC of Ciprofloxacin in two studies by Mojsoska et al. and Gupta et al., who used LB and MHB as a media, respectively, only a small difference is observed: 0.125 μg/mL vs. 0.25 μg/mL (Gupta et al., 2016; Mojsoska et al., 2021). However, in some cases such as MIC determination of Azithromycin, alterations in media can lead to drastic differences: Shen et al. used LB media and reported a MIC of 6.25 μg/mL for PAO1, whereas Bahari et al. used MHB and reported 256 μg/mL (Shen et al., 2008; Bahari et al., 2017). The examples above show that a minute change in protocol such as using different media for MIC determination impacts the outcome significantly. Consequently, the effects of sub-MIC on virulence cannot be directly compared between such studies. This observation is in line with a study from Imani Rad et al., who determined MIC of the antimicrobial agent allicin using 6 culture media including TSB, MHB, and LB. They concluded that the type of culture media significantly impacts MIC for various standard strains and that it directly influences stability of allicin (Imani Rad et al., 2017). Furthermore, it has been shown that β-lactam antibiotics can undergo rapid degradation in growth media. This highlights the need for caution when interpreting MIC values, as the actual concentration might decrease during the experiment (Brouwers et al., 2020).

Although the broth microdilution is most used to determine MIC in studies investigating sub-MICs, alternative methods exist: The disk diffusion method and the gradient method (Figures 3B,C).

Some sub-MIC studies employ the gradient method as an auxiliary method to the broth microdilution, but it is rarely seen as the only method for MIC determination (Kraupner et al., 2018). It centers on plastic strips impregnated with a predefined antibiotic concentration gradient on the underside (Jorgensen and Ferraro, 2009; Gajic et al., 2022). These strips are placed face down on an agar plate inoculated with the bacterium of interest. Following incubation, with the help of concentration markings on the top side of the strip, the MIC can be determined by the point where the growth inhibition zone intersects with the test strip (Jorgensen and Ferraro, 2009; Gajic et al., 2022) (Figure 3C). Generally, studies showed that the MIC values gathered from the antimicrobial gradient method are in good agreement with MIC values determined using the standardized microbroth dilution method (Baker et al., 1991; Citron et al., 1991; Huang et al., 1992; Jorgensen and Ferraro, 2009).

Development of the disk-diffusion method dates to the early 1940s and still represents a widely used method for accurate MIC determination today (Abraham et al., 1941; Heatley, 1944; Gajic et al., 2022; EUCAST, 2023). However, we found that the disk diffusion method is not widely used for MIC determination in sub-MIC studies. We argue that this could be because it does not yield quantitative MIC information but rather classifies pathogens into resistant, intermediate and susceptible phenotypes. While in sub-MIC studies the broth microdilution and gradient method is preferred, disk diffusion is more suitable for testing in routine clinical laboratories.

Standardized frameworks for MIC determination are in place, however, not all studies adhere to them. As demonstrated in section 3, different parameters for MIC determination inevitably contribute to contrasting results across multiple studies investigating the effect of sub-MIC antibiotics on virulence and therefore hinders the exploration of a potential VIC.

## 4 Zones below MIC have been explored and specified

Almost 50 years ago, Lorian discussed that antibiotic concentrations far below the MIC value led to morphological changes in bacteria (Lorian, 1975). In 1990, Baquero surmised that a dangerous window for emergence of microbial resistance exists (Baquero, 1990). In 2003, Drlica built on that assumption and developed an *in vitro* framework with the goal to identify microbe-antibiotic relationships that could most likely lead to emergence of resistance (Drlica, 2003; Drlica and Zhao, 2007). The pharmacodynamic model explained that selection of resistant bacteria can occur at concentrations between the MIC of the fully susceptible strain (lower limit, MIC_sus_) and the MIC of the fully resistant strain [upper limit, MIC_res_, also called mutant prevention concentration (MPC)]. The range between those established boundaries was termed the “Mutant Selection Window” (Drlica, 2003; Drlica and Zhao, 2007).

### 4.1 Minimum selective concentration

Excellent reviews thoroughly discuss that selection for resistance is happening far below MIC at sub-lethal antibiotic levels (Andersson and Hughes, 2012, 2014; Hughes and Andersson, 2012). They rightfully argue that experimental data is needed to unravel the intricacies of sub-lethal antibiotic concentration on selective pressure for resistance. To push this notion forward, they pioneered this field of research, contributing vital insights by conducting multiple keystone studies (Gullberg et al., 2011, 2014; Wistrand-Yuen et al., 2018). One of these excellent papers showed that *Salmonella enterica* evolved high-level resistance under sub-MIC selection pressure (Wistrand-Yuen et al., 2018). Most interestingly, it was demonstrated that spectra of resistance mutations differed between bacteria exposed to antibiotic levels above or below MIC (Wistrand-Yuen et al., 2018).

**Figure 4:**
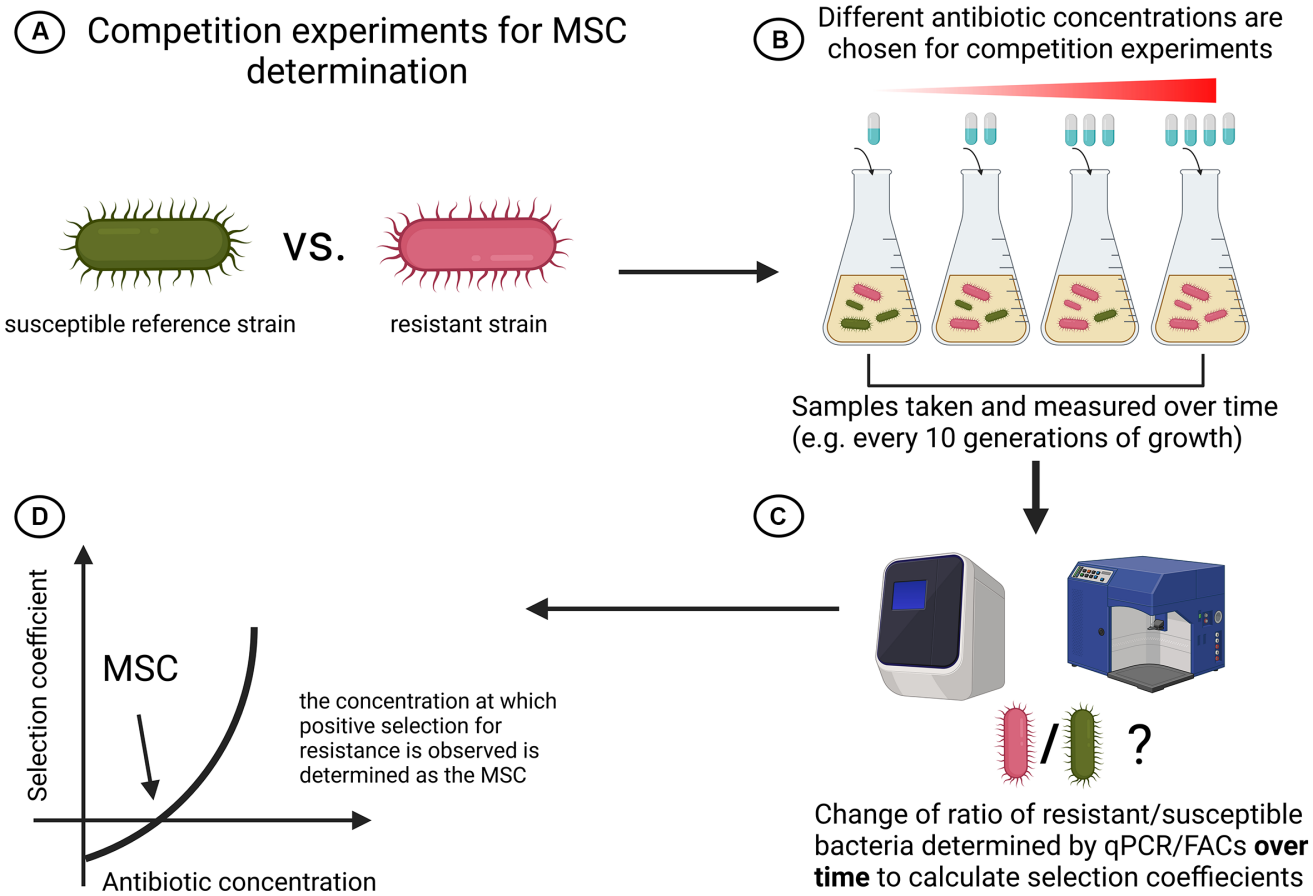
Schematic overview of simple strain competition experiments for MSC determination. **(A)** A susceptible reference strain competes with a resistant strain. To isolate effects of antibiotics seen, isogenic strains are recommended. **(B)** Both strains are cocultured and each setup is treated with a different antibiotic concentration. Samples are taken with equal spacing. **(C)** Ratio of resistant over susceptible bacteria is regularly determined with either qPCR or FACs. **(D)** The selection coefficient is plotted against the antibiotic concentration and MSC is determined as the point where the graph intersects with the x-axis (Gullberg et al., 2011). Created with BioRender.com.

Using competition experiments between isogenic pairs of resistant mutants and susceptible strains of *Escherichia coli* and *Salmonella enterica,* Gullberg et al. showed that selection of resistance occurs far sub-MIC (Gullberg et al., 2011). Fluorescent-activated cell sorting (FACs) has been used to keep track of and quantify the amount of resistant and susceptible cells over time during competition for 80 generations in various antibiotic concentrations. They calculated the selection coefficient and plotted it against antibiotic concentrations. The point at which the resistant mutant outgrew the susceptible was determined as the Minimum Selective Concentration (MSC). They found that the MSC for Streptomycin was 1/4 MIC_sus_, for Tetracycline 1/100 MIC_sus_ and for Ciprofloxacin it varied between 1/10 and 1/230 MIC_sus_ (depending on the resistance mutation) (Gullberg et al., 2011). Most interestingly, they could also show *de novo* resistant mutants can be selected for at sub-MIC concentrations (Gullberg et al., 2011). A graphical summary of the method used to determine MSC in isolated competition models can be seen in Figure 4. In contrast to this quantitative assay, a qualitative study arrived at similar conclusions using not FACs, but an elegant chromogenic culture assay to assess selection for resistance over time (Liu et al., 2011). They showed that in *Escherichia coli* 1/5 MIC of Ciprofloxacin and 1/20 MIC of Tetracycline select for outgrowth of resistant cells in competition experiments (Liu et al., 2011). Another study demonstrated that multidrug resistance plasmids are selected at antibiotic concentrations far below the MIC (Gullberg et al., 2014).

**Figure 5:**
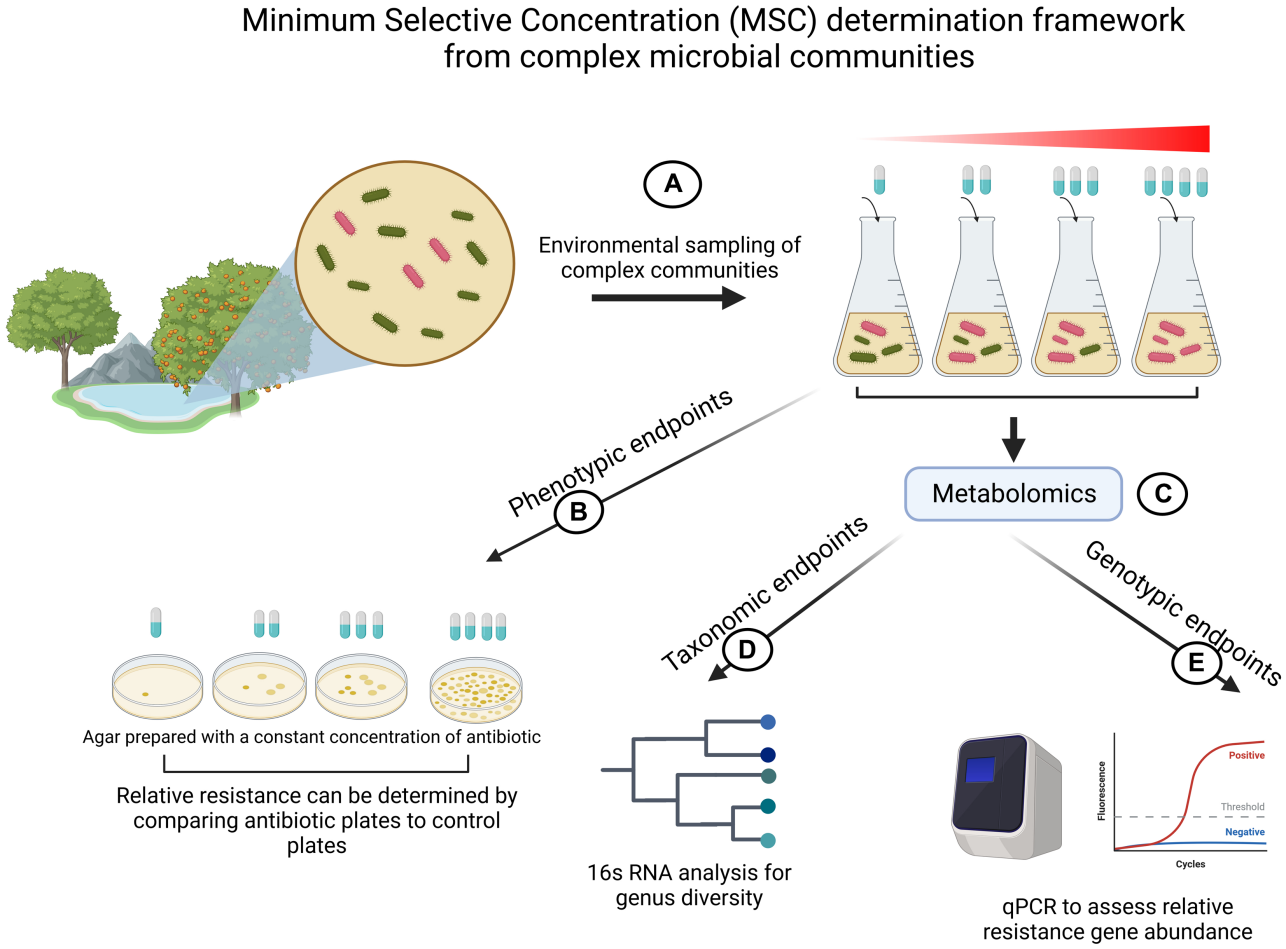
Illustration of MSC determination experimental flow for complex microbial communities. **(A)** Complex environmental samples of microbial communities are sampled and cultivated in the presence of different antibiotic concentrations. **(B)** Phenotypic profiling of resistance by counting colony forming units (CFUs) on plates prepared with a fixed amount of antibiotics. **(C)** Metabolomic exploratory assays to gain 16 s RNA data and to profile which genes respond to antibiotics. **(D)** 16 s RNA is used to explore taxonomical diversity in response to identify an antibiotic concentration which exerts selective pressure on the community. **(E)** Tracking relative abundance of resistance genes to gain insight about MSC. Created with BioRender.com.

All of the above studies provide compelling data that selection for resistance is happening at sub lethal antibiotic levels. However, one might argue that while isolated competition experiments provide valuable insight into MSC, extrapolation of the findings to entire communities is non-trivial. Hence, efforts have also been made to determine MSC not in a single species setting but in complex communities that closely represent ecosystems. These studies commonly perform high-cost metabolomic studies to define which genes increase in abundance during antibiotic exposure-response experiments. This aids experiments especially when investigating resistances that are dependent on many genes (e.g.: β-lactams) (Lundström et al., 2016). Those genes are then quantified by qPCR over time under antibiotic exposure. Next to genotypic MSC determinations, most of these studies additionally employ phenotypic methods such as counting CFUs on resistance plates or taxonomic endpoints. Figure 5 depicts a schematic overview of the existing generalized workflow to measure MSC in complex microbial environmental samples.

One of such studies used elaborate biofilm flow-through systems to assess if Tetracycline in aquatic bacterial biofilms promotes emergence of resistance by measuring phenotypic and genotypic endpoints (Lundström et al., 2016). With the help of an exploratory metabolomic assay, they identified *tetA* and *tetG* to be most significantly upregulated when stimulated with Tetracycline. By quantifying abundance of *tetG* and *tetA* with qPCR and counting CFUs they report that selection for resistance occurs at low concentration levels below MIC (≤1 μg/L), which agree with native Tetracycline concentrations in aquatic environments.

Another study followed a similar approach to calculated LOEC (lowest observed effect of concentration) and NOEC (no observed effect of concentration) of Ciprofloxacin in complex aquatic communities (Kraupner et al., 2018). They found that the most sensitive endpoints were taxonomic diversity and the gene *qnrD*, stating the NOEC in the flow through system to be 0.1 μg/L, which is identical to MSC determined by Gullberg et al. in a simplified competition experiment (Gullberg et al., 2011). The LOEC for Ciprofloxacin selection was determined to be 1 μg/L. In this study, the use of NOEC as a reasonable exposure limit for Ciprofloxacin in the environment to prevent selection for resistance is proposed (Kraupner et al., 2018).

Lastly, a study from 2018 quantified positive selection for the antibiotic Cefotaxime resistance in complex wastewater ecosystems with qPCR (Murray et al., 2018). Based on previous studies, the MSC has been profiled with qPCR and calculated by the intercepting point of selection coefficient plot with the x-axis (Gullberg et al., 2011). In line with other experiments, they proved strong positive selection of resistance genes at low, environmentally relevant concentrations (Murray et al., 2018). They could also identify a gap in MSC determination: Quantification of the antibiotic during MSC determination experiments is necessary, since in their case the antibiotic was rapidly degraded by the bacterial community (Murray et al., 2018). This means that the MSC of cefotaxime (0.4 μg/L) is likely to be even lower.

To conclude, multiple studies investigating the interplay between low levels of antibiotics and selective pressures for resistance have been conducted (Gullberg et al., 2011, 2014; Liu et al., 2011; Wistrand-Yuen et al., 2018). A handful of studies explored selection dynamics of resistance in complex microbial communities by determining genotypic, phenotypic and taxonomic endpoints (Lundström et al., 2016; Kraupner et al., 2018; Murray et al., 2018; Stanton et al., 2020). While all those studies provide essential contributions to advance research, a standardized way to determine MSC in simple and complex samples needs to be found.

### 4.2 Minimum increased persistence concentration—a zone below MSC?

Even less so than the MSC window, MIPC is a newly emerging framework that has, to the best of our knowledge, only been thematized recently (Stanton et al., 2020). They add to existing literature and proposes that a selective window below the MSC exists: The Minimum Increased Persistence Concentration (MIPC) (Stanton et al., 2020). They incubated *Enterobacteriaceae* for 7 days in Tetracycline Hydrochloride and quantified the change in gene expression of the resistance conferring gene *tetG.* In line with Lundström et al., they could see an increase in *tetG* prevalence compared to the control (Lundström et al., 2016; Stanton et al., 2020). However, when comparing the starting prevalence of *tetG* with day 7 prevalence of *tetG,* a reduction of the resistance gene could be observed. They suggest that this negative selection could be attributed to increased persistence (reduced rate of negative selection) in the pathogens (Stanton et al., 2020). They also argue that what Lundström et al. described in their paper could have been caused by increased persistence, and not as suggested by enrichment of resistance (Lundström et al., 2016; Stanton et al., 2020). Thus, they define the MIPC as a “concentration above which a significant increase in persistence is observed” and warn that it might lie below MSC (Stanton et al., 2020). The MIPC might represent an important threshold above which antibiotic concentrations lead to diminished disappearing of resistant bacteria. On one hand, this microbial enrichment would increase human exposure and overall mutation risk compared to antibiotic-free environments. On the other hand, increased persistence would lead to negative selection for resistance genes between MIPC and MSC (Stanton et al., 2020). Due to MIPC being below MSC, Stanton et al. argue that MIPC could be considered over MSC when setting antibiotic limits in the environment.

As evident in Section 4, zones below MIC have been specified and explored. When reading experimental articles about sub-MIC antibiotic treatment and its impact on virulence, a question naturally comes to mind: In which sub-MIC zone does the tested antibiotic concentration fall? All we see are concentration levels represented as fractions of MIC (e.g.: 1/2 or 1/16) but it is often unclear precisely where within the sub-MIC range a particular experiment falls. A standardized categorization of sub-MIC ranges would help to link an observed effect on virulence to a certain sub-MIC zone. This would make it easier to classify impact on bacterial virulence and thus pave the way toward targeted treatment.

## 5 What to do now?—toward a standardized framework to combat virulence

The central claim of this critical review is that the reason for ambiguous results across sub-MIC studies on virulence can in part be attributed to the absence of standardized MIC and sub-MIC methodology. To begin with, we explained the importance of having clear definitions for terms such as “persistence” and “tolerance” in sub-MIC research. Later we highlight that the lack of sub-MIC understanding already starts at the stage of MIC determination. Various examples for studies that use different MIC determination methods and therefore calculate different values for similar strains and antibiotics are given. We identified this as one of the main issues for incomparable results. Lastly, studies investigating the sub-MIC area (MSC and MIPC) are presented. Across them, consensus exists that selection for bacterial fitness traits such as persistence and resistance happens far below the MIC. While this is invaluable insight for environmental agencies to pose limitations, the connection between those findings and medical treatment possibilities needs to be made: If we gain profound understanding of sub-MIC ranges, we could characterize virulence within different ranges, opening up endless possibilities for tailored treatment opportunities.

### 5.1 Starting at the beginning—standardizing MIC determination

Perhaps not surprising, successful sub-MIC profiling calls for rigid MIC determination. Well defined frameworks such as EUCAST and CLSI are already in place, nevertheless, studies commonly deviate from those protocols when calculating MIC. EUCAST and CLSI generally recommend the broth microdilution, which agrees with the approach chosen in numerous studies. Other methods such as the disk diffusion and the gradient test are viable options, however we recommend the broth microdilution as the main method of choice for MIC determination (EUCAST, 2022; CLSI, 2023).

When performing the broth microdilution, various discrepancies can arise, with the most frequent being the use of different media (LB, MHB, TSB) between studies. This review points out the major impact the choice of growth media had on numerous study outcomes and further underlines the importance of the standardized MHB media. However, this holds only true in the case of experimental settings performed on rich, unmodified media. Experiments requiring minimal or depleted media require MIC determination in the same growth conditions. These altered growth conditions could affect the bacterial physiology, and therefore greatly modify the bacterial metabolism and antibiotic susceptibility. According to EUCAST and CLSI, the assessment of growth after antibiotic exposure should be conducted visually by the unaided eye. However, some studies used a photo-spectrometric readout. While it is difficult to infer the impact of this deviation on the outcome of various studies, it is nonetheless a step in the protocol that should be conducted according to already standardized frameworks. Moreover, it is vital to adhere to other parameters imposed by EUCAST, such as the purity of culture and the correct density of inoculum (5×10^5^ CFU/mL) (EUCAST, 2022). Lastly, other culture conditions such as time and temperature of incubation present yet another aspect of MIC determination in need of standardization.

Increasing efforts need to be focused on assessing antibiotic stability over the course of the MIC determination. While MIC tests generally presume antibiotics to be stable in growth media, degradation for antibiotics including β-lactams have been reported (Brouwers et al., 2020). One study that investigated the effect of typically used media (such as LB, MHB, TSB) found that the type of culture media influenced the stability and subsequently the MIC of the antibacterial agent allicin (Imani Rad et al., 2017). Efforts should be directed on further understanding dynamics of antibiotics in various media to avoid possible MIC determination biases and, subsequently, inaccurate sub-MIC investigation. When MIC has been calculated according to the standardized broth microdilution, sub-MIC studies usually employ double dilutions to calculate sub-MICs (e.g.: 1/2, 1/4, 1/8…). Since virulence is impacted by minute changes in antibiotic concentration, it would me more insightful to do arithmetic dilutions for more reliable and granular results. Should diffusion tests be used, measuring colony size by imagine-scanning techniques could prove useful to measure sub-inhibitory effects.

### 5.2 How to standardize sub-MIC zones?

Recently, zones below MIC such as MSC and MIPC have been described (Gullberg et al., 2011, 2014; Liu et al., 2011; Andersson and Hughes, 2012, 2014; Hughes and Andersson, 2012; Stanton et al., 2020). In contrast to MIC determination, no agreed guidelines and methodologies to profile sub-MIC for a given organism exist.

Recent endeavors to develop methods for determination of MSCs of antibiotics present an excellent start and efforts toward understanding selective pressures on microbial traits have been made in recent years. Although the body of research in this area is limited, intriguing similarities, but also limitations of experimental intricacies can be observed.

MSC determination experiments can be conducted based on Gullberg et al. in simplified two strain competition experiments: There, general consensus is to track the ration of tagged (e.g.: fluorescent) susceptible and resistant isogenic strains in a co-culture (Gullberg et al., 2011). The antibiotic concentration at which the resistant mutant outgrows the susceptible one is termed MSC (Figure 4). Such systems could readily be standardized in many ways: First, it is of utmost importance that for every pathogen a susceptible reference strain against which the pathogen of interest is competing, needs to be found. Second, as in MIC determination, the media in which the experiment takes place must be standardized and investigated for potential antibiotic degradation to rule out biases. Third, guidelines for parameters of the competition like number of serial passages, overall duration of the experiment and time of sampling must be standardized. Lastly, a common readout method to track the ratio of the competing cells must be found (FACs, qPCR). The implementation of standardized guidelines to test for MSC with strain competition experiments in clinical settings is an important step that needs to be taken. However, the method also comes with some limitations that need to be addressed: The competing strains used by past studies are isogenic with the only exception being the fluorescent tags by which their abundance can be tracked. This presents an issue, since clinical isolates are most probably not isogenic, making it hard to attribute the effect we see solely to the antibiotic. Nonetheless, we argue that it would still give an estimate of the area in which selection for resistance might occur.

Simple strain competitions are vital and should be readily standardized in clinical contexts. However, it also becomes clear that findings might not be translatable to bigger microbial communities and, ultimately, with the aim of treatment, to *in vivo* environments. A limited number of studies investigating MSC of microbial communities have been conducted (Lundström et al., 2016; Kraupner et al., 2018; Murray et al., 2018; Stanton et al., 2020). However, thus far, the focus of MSC research has primarily been on exploring its application in determining environmental regulation thresholds. Analysis of such studies for this review revealed a consensual workflow that could potentially be translated to sub-MIC virulence profiling and treatment opportunities (Figure 5). Most studies measure MSC for various types of endpoints, all of which should follow agreed guidelines: Phenotypic endpoints to assess MSC in environmental communities presents invaluable information in case not all genes that contribute to resistance are known. CFU counts could present a method that could easily be standardized in terms of media used and number of bacteria plated.

Furthermore, most studies investigating selective properties of antibiotics in environmental communities utilize genotypic endpoints. Before measuring the endpoints, metabolomic assays such as shotgun-sequencing are employed to find genes that are impacted by antibiotic exposure. Subsequently, the relative abundance of these genes is then quantified using the gold-standard qPCR assay. Due to the high cost of large-scale metabolomics, we propose the following: In order to standardize this workflow, exploratory metabolomic data should be available for everyone, preferably in a large database that shows up and downregulated genes across multiple species of communities under exposure of antibiotics. That way the assay does not need to be done every time and a core list of crucial genes can readily be analyzed with qPCR. As argued by Stanton et al., it is also necessary to assess the starting prevalence of genes. This would help assess if the genes are under positive or negative selection and one could therefore differentiate between resistance and persistence mechanisms (Stanton et al., 2020). When establishing general protocols for genotypic endpoint measurements, this is something that needs to be considered.

However, Kraupner et al. warn that changes in gene abundance should not always be taken at face value since they could also be consequences of taxonomic shifts (Kraupner et al., 2018). Taxonomic analysis represents another intriguing way that has been used to study MSC and should be considered when profiling a microbial community. For this, a diversity analysis to identify an antibiotic concentration which exerts selective pressure on the community could be employed.

For a successful MSC profiling of complex communities, investigation of phenotypic, genotypic and, possibly taxonomic endpoints are essential. A rigid guide exploring phenotypic and genotypic MSC determination should readily be standardized for rapid profiling of complex communities. Taxonomic analysis presents a new way to look at the whole picture but due to its steep costs and need of specific equipment it does not need to be prioritized when outlining standardized protocols.

The newly emerging concept of a sub-MSC zone, the MIPC zone presents another thought-provoking concept. To the best of our knowledge, only one study came up with this term and investigated the matter (Stanton et al., 2020). Between the MSC and the MIPC, they explain that the number of resistant bacteria could be higher than if there was no antibiotic present (Stanton et al., 2020). They highlight that selection happens below the MSC level and that we need to look even deeper to fully profile pathogens. Future work needs to be focused on fully characterizing persistent phenotypes and understanding the underlying epigenetic mechanisms. qPCR endpoints for generalized pathogen- specific set of genes accountable for persistence then need to be analyzed for reduced rate of negative selection under antibiotic exposure.

### 5.3 Transition to *ex vivo* microbial community studies could open the door for targeted therapies

As discussed earlier, isogenic strain experiments could present an integral part of clinical diagnostics and profiling of single strain pathogens against a reference strain. But as the step toward clinical profiling of larger microbial communities needs to be made, community assays discussed in this review present a solid base to build upon. They already have a good framework in place and could be used to successfully profile complex human communities from phenotypic, genotypic and possible taxonomic angles. For this, standard media best representing the *in vivo* environment needs to be established.

With a proper sub-MIC profiling in place, the impact of antibiotics on virulence could be systematically unraveled and a VIC of a specific antibiotic could potentially be determined more accurately for a given pathogen. This brings up the last issue briefly discussed in this review: How should virulence be measured? Virulence presents a complex topic and is highly strain specific. Every pathogen has different ways of causing harm to the host, and many ways to measure it exist. However, going over the methodology to assess virulence in main pathogens lies outside the scope of this paper. Nonetheless, we propose that when characterizing a number of clinical isolates, it is crucial to profile specific virulence molecules for a virulence signature against a reference strain.

The overarching claim of this review was that the challenge of investigating impact of sub-MIC antibiotics on virulence lies in part in the absence of uniform MIC and sub-MIC profiling. This review highlights topics that need to be regulated and gives suggestions on how to build a framework for successful profiling. It identifies MIC determination as one of the main culprits in need for standardization. It explains that experimental frameworks for competition experiments and environmental community analysis should be built upon to extend their applicability to clinical settings. Consensus in virulence measurements combined with a full sub-MIC profiling would pave the way for potential targeted treatment and would allow us to better understand how virulence manifests itself in the sub-MIC world.

## Author contributions

FT: Data curation, Formal analysis, Investigation, Visualization, Writing – original draft, Writing – review & editing. FA: Conceptualization, Funding acquisition, Investigation, Project administration, Resources, Supervision, Visualization, Writing – review & editing.
